# NGS-dataset of putative driver mutations associated with benign peritoneal strumosis

**DOI:** 10.1016/j.dib.2018.08.006

**Published:** 2018-08-16

**Authors:** Michael Brockmann, Verena Schildgen, Oliver Schildgen, Jessica Lüsebrink, Monika Pieper, Alexandru Gudima

**Affiliations:** a Kliniken der Stadt Köln gGmbH, Klinikum der Privaten Universität Witten/Herdecke, Institut für Pathologie, Ostmerheimer Str. 200, D-51109 Cologne, Germany; b IMSP Institutul Oncologic din Republica Moldova, Sectia Ginecologie nr.2, str. N. Testemitanu 30, MD-2025 Chisinau, Republic of Moldova

## Abstract

A rare case of benign peritoneal strumosis was screened for driver mutations in genes relevant to currently approved cancer therapies. Therefore, three formalin fixed paraffin embedded issue sections were screened with the GeneReader Actionable Insights NGS panel (Qiagen, Hilden, Germany) for the occurrence of driver mutations. Several mutations were identified in drug-targetable genes, such as ALK, EGFR, and BRAF. The majority of identified mutations were single nucleotide variant, but also a insertion/deletion mutation was identified. The presented dataset is the first NGS dataset available from a patient with benign peritoneal strumosis.

**Specifications Table**

**Table:** 

Subject area	*Medicine, clinical and basic tumor research*
More specific subject area	*Benign peritoneal strumosis*
Type of data	*Table*
How data was acquired	*Next Generation Sequencing (NGS) with Qiagen GeneReader Actionable Insights Panel*
Data format	*Analyzed data, comprehensive*
Experimental factors	*DNA extraction from formalin-fixed paraffin embedded slides, NGS*
Experimental features	*NGS with Qiagen GeneReader Platform according to manufacturer׳s recommendations*
Data source location	*Clinical case from Republic of Moldova, investigated in Cologne, Germany*
Data accessibility	*All data are included in this Data in Brief article*
Related research article	*No related Research Article*

**Value of the data**

• The knowledge on molecular alterations occurring in benign peritoneal strumosis is limited, the present data extent this limited knowledge.
• The presented data could trigger further (multicenter) studies on benign strumosis.
• The data show that no benign peritoneal strumosis can occur without “classical” targetable mutations.

## 1 Data

A dataset obtained by next generation sequencing of drug-targetable genes of a clinical case with benign peritoneal strumosis is presented. Benign peritoneal strumosis is a clinical condition that is rarely observed [1] and so far was not characterized on a molecular level.

The dataset was obtained from histologically confirmed formalin fixed paraffin embedded tissue samples of a 33 year old Caucasian woman with benign peritoneal strumosis. To date there were no datasets available that reported the mutational landscape of drug-targetable genes in clinical cases of benign peritoneal strumosis.

We identified several mutations that to date are classified as mutations with uncertain pathogenic role (Table 1). In particular, we observed the single nucleotide variation (SNV) c.4381A>G (p.Ile1461Val) mutation in the ALK gene, the c.1822delC (p.His608fs) mutation in the BRAF gene, the c.1562G>A (p.Arg521Lys) mutation in the EGFR gene, the c.1963A>G (p.Ile655Val) and the c.3508C>G (p.Pro1170Ala) mutation in the ERBB2 gene, the c.386A>G (p.His129Arg) in the ERBB3 gene, as well as the c.291–10delT deletion in the KRAS gene.

**Table 1:** Overview on the mutations detected by NGS in tissue samples of a patients with benign peritoneal strumosis.

**Gene**	**c. variant**	**p.variant**	**Type**
ALK	c.4338C>T	–	SNV
ALK	c.2535T>C	–	SNV
ALK	c.4381A>G	p.Ile1461Val	SNV
BRAF	c.1882delC	p.His608fs	Deletion
PIK3CA	c.-76-14537C>G	–	SNV
PDGFRA	c.1701A>G	–	SNV
KIT	c.2362-77G>A	–	SNV
EGFR	c.474C>T	–	SNV
EGFR	c.2361G>A	p.Arg521Lys	SNV
EGFR	c-1881-781C>T	–	SNV
EGFR	c.2361G>A	–	SNV
ERBB2	c.1963A>G	p.Ile655Val	SNV
ERBB2	c.3508C>G	p.Pro1170Ala	SNV
ERBB3	c.386A>G	p.His129Arg	SNV

Dataset of mutations identified in benign peritoneal strumosis analyzed by NGS.

## 2 Experimental design, materials, and methods

In order to identify putative therapy targets and to further characterize this rare clinical condition we have performed a Next Generation Sequencing (NGS) analyses using the GeneReader Actionable Insights panel (Qiagen, Hilden, Germany) as recently described [2], [3]. Briefly, DNA from three different FFPE tumor tissue samples from the patient was isolated with the Maxwell FFPE kit (Promega, Darmstadt, Germany) according to the manufacturer׳s protocol. The DNA samples were then processed for sequencing with the GeneRead QIAact Actionable Insights Tumor Panel strictly according to the manufacturer׳s recommendations as follows. The GeneRead QIAact Actionable Insights Tumor Panel was used for target enrichment of ALK, BRAF, EGFR, ERBB2, ERBB3, ESR1, KIT, KRAS, NRAS, PDGFRA, PIK3CA, and RAF1. Following target enrichment, library preparation and clonal amplification was done using the GeneRead DNA Library Q Kit and the GeneRead Clonal Amp Q Kit. DNA quality and concentration was determined after target enrichment and library preparation with the QIAxcel DNA High Resolution Kit on the QIAxcel instrument. Sequencing was performed on the GeneReader instrument with the GeneRead Sequencing Q Kit. Qiagen Clinical Insight Software was used for analysis and interpretation of the results. Sequencing products are analyzed by comparison with the following reference sequences: NM_004304.4 (ALK), NM_004333.4 (BRAF), NM_005228.4 (EGFR), NM_004448.3 (ERBB2), NM_001982.3 (ERBB3), NM_001122742.1 (ESR1), NM_000222.2 (KIT), NM_004985.4 (KRAS), NM_002524.4 (NRAS), NM_006206.5 (PDGFRA), NM_006218.3 (PIK3CA), and NM_002880.3 (RAF1).
